# A pan-genome method to determine core regions of the *Bacillus subtilis *and *Escherichia coli* genomes

**DOI:** 10.12688/f1000research.51873.2

**Published:** 2021-09-02

**Authors:** Granger Sutton, Gary B. Fogel, Bradley Abramson, Lauren Brinkac, Todd Michael, Enoch S. Liu, Sterling Thomas

**Affiliations:** 1 J. Craig Venter Institute, Rockville, Maryland, 20850, USA; 2 Natural Selection, Inc., San Diego, CA, 92121, USA; 3 Noblis, Inc., Reston, VA, 20191, USA

**Keywords:** pan-genome, pan-genome graph, core genes, essential genes

## Abstract

**Background:** Synthetic engineering of bacteria to produce industrial products is a burgeoning field of research and application. In order to optimize genome design, designers need to understand which genes are essential, which are optimal for growth, and locations in the genome that will be tolerated by the organism when inserting engineered cassettes.

**Methods:** We present a pan-genome based method for the identification of core regions in a genome that are strongly conserved at the species level.

**Results:** We show that the core regions determined by our method contain all or almost all essential genes. This demonstrates the accuracy of our method as essential genes should be core genes. We show that we outperform previous methods by this measure. We also explain why there are exceptions to this rule for our method.

**Conclusions:** We assert that synthetic engineers should avoid deleting or inserting into these core regions unless they understand and are manipulating the function of the genes in that region. Similarly, if the designer wishes to streamline the genome, non-core regions and in particular low penetrance genes would be good targets for deletion. Care should be taken to remove entire cassettes with similar penetrance of the genes within cassettes as they may harbor toxin/antitoxin genes which need to be removed in tandem. The bioinformatic approach introduced here saves considerable time and effort relative to knockout studies on single isolates of a given species and captures a broad understanding of the conservation of genes that are core to a species.

## Introduction

The primary focus of this paper is a new pan-genome method to determine core regions of a genome shared by all or almost all strains of the same species or subspecies. We evaluate the performance of this approach relative to other methods using experimentally determined essential genes under the hypothesis that all or at least most essential genes should be core across a species. This hypothesis implies that methods for determining core regions/genes are likely to be more accurate if they identify more essential genes as core genes. The paper reveals the potential usefulness of pan-genome analysis for synthetic engineering and genome analysis more broadly through the analysis of core regions in *Bacillus subtilis* and *Escherichia coli*.

Over the last decade, considerable interest has been directed towards the determination of a minimal bacterial cell, making use of a short genome consisting of only essential genes for viability. The *Mycoplasma mycoides* JCVI-syn3.0 is a case example of synthetic engineering to design and build a genome that contains a streamlined gene set essential for cell viability and cell replication. ^1^ Multiple genome reduction projects have been undertaken. ^2– 4^ More targeted genomic deletions of genomic loci have been performed to characterize essential genes, but generally targeted approaches are too laborious to perform on a whole genome. ^5, 6^ However, the identification of “essential” genes - those genes that are critical for cell viability and replication - takes considerable time and effort in a laboratory setting and is usually determined with respect to one reference genome under one set of specific growth conditions. For instance, Kobayashi *et al.* ^7^ and Koo *et al.* ^8^ experimentally and computationally determined the minimal gene set in the Gram-positive bacterium *Bacillus subtilis.* Koo *et al.* ^8^ used a strictly experimental approach but Kobayashi *et al.* ^7^ used three forms of evidence for their essential genes as given in their Table 4: RB: previous experimental work in *B. subtilis*; RO: previous experimental work in other bacteria; and TW: their experimental work. The RO evidence used is a mix of experimental and computational as the determination of orthologs is computational and essentiality of those orthologs was not experimentally confirmed: “Through predictions we propose that 79 other genes are essential, whereas 106 are not (Table 3)”. ^7^ Of the ~4,100 genes of the type strain, a total of 271 genes for Kobayashi *et al.* ^7^ and 257 genes for Koo *et al.* ^8^ were shown to be essential. These essential genes were further categorized in terms of cell metabolism and enzymatic capability. Additionally, for the ~4400 genes in the Gram-negative bacterium *Escherichia coli*, Goodall *et al*., ^9^ Baba *et al.*, ^10^ and Yamazaki *et al.*, ^11^ it was determined that 414 genes were essential to strain K-12.

Reuß *et al.* ^3^ completed extensive further experimental and computational work to determine a minimal *B. subtilis* genome they call *MiniBacillus.* They present a list of 523 protein coding and 119 RNA genes necessary for a minimal *B. subtilis* cell growing in complex medium at 37°C. While many of these genes are not essential under single deletion experimental conditions, they are required for survival because a cell needs certain essential functions which may be carried out independently by more than one gene. As noted by Reuß *et al.*, ^3^ the choice of which functionally isologous genes to choose for a minimal cell depends upon minimization goals and gene choices for different functions are not independent of one another. One criterion used by Reuß *et al.* ^3^ is the conservation of the gene: “More strongly conserved genes were preferred over less conserved genes. In this respect, gene conservation and essentiality in genome-reduced *Mycoplasma* and other mollicutes species and the inclusion of genes in the genome of *M. mycoides* JCVI-syn3.0 had a high priority”. Reuß *et al.* ^3^ do not explicitly use gene conservation at the species/subspecies pan-genome level but this seems in spirit with their criteria.

Reuß *et al.* ^3^ extended their computational prediction of *MiniBacillus* by building on previous work to generate *B. subtilis* strains with large genome reductions. ^2^ They started by constructing the delta 6 strain ^12^ (~8% genome reduction). This reduction removed: “two prophages (SPβ, PBSX), three prophage-like regions, and the largest operon of *B. subtilis* ( *pks*).” The phage/prophage regions were identified in part by GC content and codon usage as a method to identify probable horizontally transferred regions. Pan-genome analysis was not used. Next, strain IIG-Bs20 was constructed from delta 6 ^13^ by removing “all nine prophages, seven antibiotic biosynthesis gene clusters and two sigma factors for sporulation” in part to have a strain that would “not produce spores, antibiotics or bacteriocins”. A direct descendant of IIG-Bs20, strain IIG-Bs27-47-24 (~31% genome reduction), was then used to generate more reductions in two independent strains, PG10 (~35% genome reduction) and PS38 (~36% genome reduction) ^2^ with the goal of removing genes “not necessary for the survival of the cell ( *e.g.*, sporulation, antibiotic production, motility, metabolism of secondary carbon sources, and genes of unknown functions)”. For the strains IIG-Bs27-47-24, PG10, and PS-38, pan-genome analysis was not explicitly used but one of several criteria for deleting genes was a lack of conservation across broader taxonomic groups.

For *E. coli*, Kolisnychenko *et al.* ^5^ generated an initial reduced genome in order to “serve both as a better model organism and as a more useful technological tool for genome science” by “deleting the largest K-islands of *E. coli*, identified by comparative genomics as recent horizontal acquisitions”. K-islands are regions unique to the K-12 strain MG1655 compared with the O157:H7 strain Sakai, and the uroseptic *E. coli* strain CFT073. This comparative analysis with a limited set of genomes is an obvious precursor to pan-genome analysis with a much larger set of genomes. Umenhoffer et al. ^14^ generated the reduced *E. coli* strain MDS42 to be “free of mutation-generating IS elements”. This approach does not rely on comparison to other strains, just the ability to identify IS elements. Csorgo *et al.* ^15^ further reduced the MDS42 strain by “constructing low-mutation-rate variants … to lack most genes irrelevant for laboratory/industrial applications.” They targeted genes likely to be core and necessary for the species to adapt to the environment but detrimental in an industrial setting where strain stability is important.

Experimental studies to determine such essential genes are time consuming and often restricted to a single environmental condition using a single strain of the species. In addition, these approaches also knock out one gene at a time. As such, genes with multiple copies with redundant functions are often not considered as essential following knockout, as their additional copy is able to maintain cellular function. In other words, a viable organism would not result from deleting all but the experimentally determined essential genes from the genome. Another peculiarity of the single knockout essential genes is that pairs or cassettes of genes which can be removed and still have a viable organism are labeled essential because removal of just one gene is lethal. For example, removing the methylation gene(s) without removing the restriction digestion enzyme genes from the restriction mechanism results in cell death but the cell survives if the entire system is removed. This is likewise true for toxin/antitoxin systems.

While it is possible to define “essential” genes relative to viability, another larger question remains; which genes define a species? While specific phenotypes can vary across strains, in general a species seems to require some minimal set of genes to not only survive in the laboratory but to thrive in its natural environment. In contrast, some strains may have retained or acquired some genes which improve survival for specific niches. Comparing the genes from multiple diverse strains of a species can help answer these questions. We define the pan-genome for a species/subspecies to be the set of predicted orthologous gene clusters (OGC) across that set of strains. Others have allowed paralogs to be included in these gene clusters ^16– 18^ but here we do not. This constraint forces there to be at most one gene per genome in an OGC.

We further define a pan-genome graph (PGG) to be a graph with the pan-genome OGCs as nodes where an edge exists between two nodes if the respective genes for any genome from the two OGCs are adjacent in that genome. More precisely, an OGC node is represented as a dipole with 5′ and 3′ ends and the edges go between an end of one node (5′ or 3′) to an end of another node depending on the orientation of the genes which are adjacent. The edges primarily represent the order and orientation of OGCs in the pan-genome genomes. Secondarily, the edges also represent the interstitial DNA sequences between the genes. A PGG edge has a weight equal to the number of genomes which contain the indicated adjacent gene ends. Core OGCs are defined to be those OGCs present in some large percentage of the strains in the pan-genome (≥95% in this work). Core edges are defined similarly. Core regions are defined to be the coordinates in a genome for each set of adjacent core genes in that genome provided the edges between the core genes are also core edges.

The PGG is important as it captures the structure of the pan-genome in ways that simply treating the pan-genome as a set of OGCs cannot. The inherent gene context in the PGG allows for more accurate annotation of a novel genome than OGCs alone which struggle to differentiate recent paralogs/repeats. The PGG allows core regions to be defined for any genome rather than just core OGCs/genes. The PGG indicates which OGCs occur in cassettes with implications for function, evolution, and synthetic engineering. As discussed later, the PGG allows for determination of probable orthologs not captured in the OGCs.

Core OGCs should determine the baseline phenotype (capabilities and traits) of a species. Previous pan-genome studies ^19^ have shown that species tend to only tolerate the placement of noncore genes between core regions and not within those core regions. The reason an organism might constrain a core region rather than just core OGCs is that the region may include regulatory mechanisms such as operons, which allows for co-expression of multiple functionally associated genes, or regulons which would be disrupted with the insertion of other genes. We believe that conservation of core regions in species indicates resistance to insertion or deletion of genes in these regions through evolution or through human-mediated genetic engineering.

**Figure 1.**
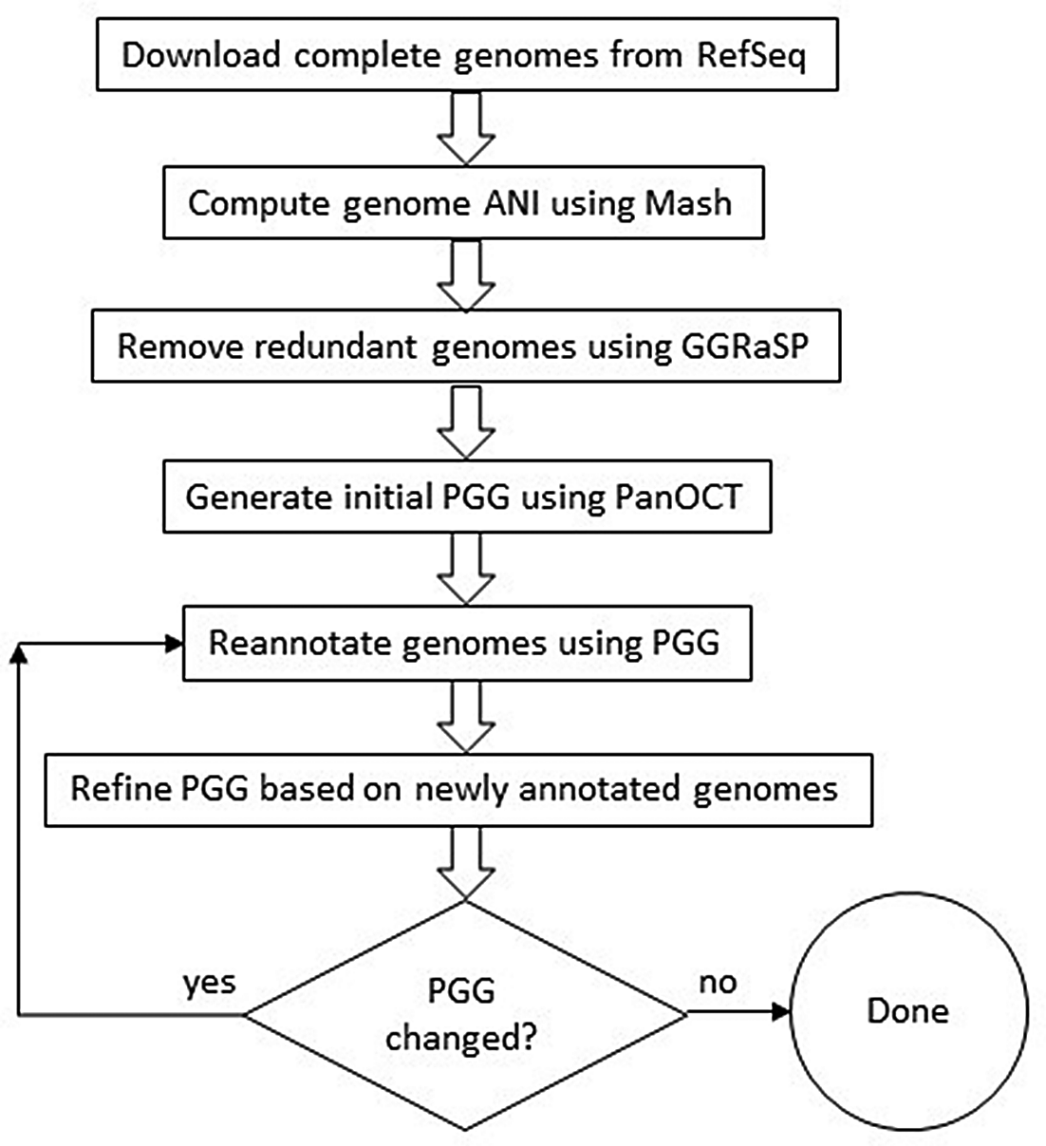
High-level overview of our method for generating a refined PGG.

Here we present a pan-genome based calculation of core regions for *B. subtilis* ssp. *subtilis* and for *E. coli.* These core regions are compared with previous experimentally determined essential genes from the literature. These core regions are not a replacement for experimentally determined essential genes, but rather provide complementary information about a much larger portion of the genome. We expect that all truly essential genes for the species/subspecies would be a subset of the core OGCs/regions, since core OGCs would encompass genes responsible for providing a fitness advantage in environmental conditions as well as being essential for viability. This approach automates computational prediction of core OGCs/regions which can be used to help guide the removal of genome regions not needed for species fitness and indicate which genome regions are amenable to engineered insertions. This approach is an incremental improvement over previous computational methods to aid genome engineering. Ortholog prediction ^17, 18, 20^ and determination of genes essential for most bacteria has a long history. ^21, 22^ Computational prediction of nonessential genes *via* predicting prophage regions or other horizontal transfer events is also well established. ^23– 25^ Pan-genome tools, most of which at some level predict core genes, are also not new. ^26, 27^ Our method builds directly upon our previous pan-genome work ^28– 30^ and includes several improvements: 1) being able to use only complete high-quality genomes (this concept is not new, but we find it impacts the quality of the PGG and core region determination and is reasonable as more complete genomes become available); 2) checking for including the correct species/subspecies using average nucleotide identity (ANI); 3) reannotating gene features using homology and gene context to ensure consistency; and 4) generating a PGG for a rigorous definition of a core region. Figure 1 shows the high-level view of our method with details provided in the Methods section.

## Methods

### Genome Selection

Reference *B. subtilis* ssp. *subtilis* and *E. coli* genomes were selected for pan-genome construction using a series of filtering steps resulting in high-quality, non-redundant genome datasets ( Table 1). For *B. subtilis* ssp. *subtilis* and *E. coli*, we selected strains with complete genomes in RefSeq. ^31^ We restricted our analysis to complete genomes to ensure that missing genes due to incomplete genome sequencing/assembly did not affect the approach or results. We limited our choice to RefSeq for two reasons: RefSeq performs a series of quality checks to remove dubious genome assemblies, and the initial pan-genome construction depends upon reasonably consistent annotation which RefSeq provides. We extracted the genomes based on organism name: *Bacillus subtilis* (we did not specify subspecies, since for many RefSeq genomes a subspecies is not given) and *Escherichia coli* (we also specified *Shigella* since all *Shigella* species are actually considered to be the same species as *Escherichia coli*). ^32, 33^

For each pan-genome, we then compared the genomes using a fast Average Nucleotide Identity (ANI) estimate generated using the MASH distance subtracted from 1 and multiplied by 100. ^34^ We used type strains and ANI to determine which of these genomes were the desired organism. We also used ANI to remove very closely related strains to reduce oversampling bias (for example for the *B. subtilis* type strain, 168, has at least eight genomes in RefSeq). We used GGRaSP ^28^ to choose a single medoid sequence from any complete linkage ANI cluster with a threshold of 0.01% or 1/10,000 base pair difference. We remove all other genomes besides the medoid as being redundant. Each removed redundant genome would be ≥99.99% ANI to the retained medoid genome. The strain 168 medoid genome is the Entrez reference genome for the *B. subtilis* type strain (GenBank sequence AL009126.3, BioSample SAMEA3138188, Assembly ASM904v1 **/**GCA_000009045.1) which can be used to map the Kobayashi *et al.* ^7^ and Koo *et al.* ^8^ results.

Using this approach, for *B. subtilis*, 143 genomes were downloaded from RefSeq. Of these, 132 genomes were determined to be *B. subtilis* ssp. *subtilis* based on type strains and ANI. The minimum ANI between any pair of the 132 *B. subtilis* ssp. *subtilis* genomes was 97.28% whereas the maximum ANI of any of the 11 other genomes to the 132 genomes was 95.73%, providing good separation between the other subspecies. By sorting the pairwise ANI matrix rows based on the ANI values in the type strain column it was clear there was a punctate threshold at ~96.5% ANI which divided *B. subtilis* ssp. *subtilis* genomes from other genomes. This means the 11 removed genomes all have ≤ 95.73% ANI to the type strain well below the 96.5% ANI threshold. The 132 genomes were further reduced to 109 genomes after removing redundant strains (using GGRaSP as discussed above). Finally, we removed strain delta6 (BioSample SAMN05150066) because it is known to have been engineered to remove multiple genes. Thus, we were left with 108 *B. subtilis* genomes ( Table 1). For *E. coli* (and *Shigella*) we downloaded 1097 complete genomes from RefSeq. Of these, 1096 were determined using ANI to be *E. coli.* The non- *E. coli* genome was clearly mislabeled as its maximum ANI to any other genome was 82.27%.

The minimum pairwise ANI of any of the 1096 genomes was 95.53% which is not as tight as for *B. subtilis* ssp. *subtilis* which is to be expected given that *E. coli* is a species grouping not a subspecies grouping. One could arbitrarily try to choose a tighter grouping around the K-12 reference genome but the pairwise ANI values of the other genomes compared with the K-12 reference genome vary continuously from 96.22% to 100% with no punctate break in the values. After removing redundant genomes (using GGRaSP as discussed above), 969 *E. coli* genomes remained. We added back in two redundant genomes: The K-12 Entrez *E. coli* reference strain MG1655 (BioSample SAMN02604091) and the K-12 strain BW25113 (GenBank sequence accession CP009273.1, GenBank Assembly accession ASM75055v1/GCA_000750555.1, GenBank BioSample accession SAMN03013572) used by Goodall *et al*. ^9^ These two redundant genomes were added back in so that we could map the PGG OGCs to these genomes for comparison to the established literature resulting in 971 genomes in the PGG ( Table 1). By using a 95% threshold for the number of genomes an OGC must be in to be considered core, some small number of the 971 genomes could be engineered to remove what are normally core OGCs and not affect the assignment of core OGCs.

**Table 1.** Number of *B. subtilis* and *E. coli* genomes selected after each genome filtering step.

Organism	Text-based query RefSeq download	ANI classification	GGRaSP redundancy filtering	Final genome dataset
*B. subtilis*	143	132	109	108
*E. coli*	1097	1096	969	971

### Pan-genome and PGG construction

For *B. subtilis* ssp. *subtilis* and *E. coli*, initial pan-genomes were based on the RefSeq annotation of these genomes. The pan-genome was generated using the pan-genome pipeline at the J. Craig Venter Institute (JCVI) at the nucleotide level using default parameters with the exception that a minimum of 90% identity and 90% length for pairwise BLAST matches were used to prevent possible clustering of non-orthologous genes. ^29^ This produced OGCs using gene context ^30^ as well as a PGG. ^19^ The PGG has two main components: nodes representing OGCs, and edges representing the sequence between OGCs and the order and orientation of the OGCs in the genomes. We updated the code repository for the JCVI pan-genome pipeline with a script: iterate_pgg_graph.pl, which calls pgg_annotate.pl for the genomes in the existing PGG in order to ensure consistent annotation of the genomes and iterates until the PGG stabilizes. The script pgg_annotate.pl uses an existing PGG to assign regions of a genome to nodes of the graph. This is done by searching the medoid sequence using BLAST for the OGC the node represents against the genome and then uses Needleman – Wunsch ^35^ to extend the alignment if needed. If there are conflicting BLAST matches, then the matches are resolved based on which matches are consistent with the structure of the PGG which encapsulates gene context across the entire pan-genome. Once the nodes of the PGG are mapped to each of the genomes in the pan-genome a new version of the PGG is intrinsic and then explicitly extracted. This process is iterated to stability. This ensures that each genome is consistently annotated so that genes missing from the original annotation of some genomes will be consistently annotated across all genomes. A user manual for this new functionality is available at https://github.com/JCVenterInstitute/PanGenomePipeline.

Core regions were determined based on the PGG. Nodes in the PGG were OGCs. Edges in the PGG represented adjacency of genes (contained in the OGCs) in the underlying genomes. The definition of which OGCs were or were not considered “core” was determined relative to a threshold criterion. We used a criterion for core such that 95% or more of the underlying genome had to contain the OGC or edge. Considering that we used only complete genomes it might have been possible to use a 100% threshold. However, we opted for a 95% threshold based on prior experience and an abundance of caution to not under call core OGCs/edges which might result in false negatives. Each core region began with a core OGC followed by a core edge (if possible, otherwise the core region comprises a single OGC) to another core OGC and so on until a core edge cannot be found to continue the core region. A core region is just a path in the PGG which was then mapped onto each genome to determine the core region coordinates. When the core threshold was below 100% any genome may be missing an OGC (gene) or edge along this path which results in the path being broken into its remaining constituent parts.

### Comparison to essential genes

In order to compare core regions to experimentally determined essential genes we needed a common base of reference. For each of the experimental studies, the genes are specified based on a reference strain that was used for the experiments and has a complete genome in RefSeq. For Kobayashi *et al*., ^7^ only gene symbols/names were given which we mapped to Entrez GeneIDs using Entrez search. GeneIDs with no matches were manually curated to estimate the best matching gene symbol listed in the literature. For Koo *et al.*, ^8^ locus IDs were provided giving direct access to the gene coordinates for RefSeq accession NC_000964.3 (BioSample SAMEA3138188, Assembly GCF_000009045.1). For Goodall, we used the data from three studies in Table S2 from Goodall *et al*. ^9^ Gene symbols/names again were all that was available but these were consistent with the GenBank annotation downloadable in gff format for the K-12 BW25113 reference genome (GenBank accession CP009273.1) used by Goodall *et al.* ^9^ (BioSample SAMN03013572). This gave us coordinates for all essential genes on RefSeq genomes which were annotated with a PGG which produces a file with coordinates for OGCs and edges mapped to the genome. These coordinates allow us to affiliate essential genes to OGCs.

## Results

The original and refined PGG statistics for *B. subtilis* and *E. coli* are provided in Table 2. The major goal of refining the PGG using reannotation and iteration until stabilization was to achieve consistent annotation across all genomes in the PGG leading to a more comprehensive and cohesive PGG. While the RefSeq annotations of these genomes tends to be highly consistent, many small genes are often arbitrarily called from genome to genome and even some common longer genes can occasionally be missed. There are three obvious points of improvement in the refined PGG for both the OGC and edge stats: the number of size 1 OGCs/edges significantly decreased due to some dubious RefSeq gene calls being eliminated and some becoming shared with other genomes; the number of core OGCs/edges significantly increased showing an improvement in the consistency of annotation across all genomes; and the number of genes/edge instances in OGCs/edges greatly increased again indicating a much more consistent annotation. We have included core OGC statistics for three threshold definitions of core: 95%, 99%, and 100%. In part, this is for comparison to previous studies but it also illustrates the relative larger impact of consistency as the threshold increases. For example, in *E. coli*, the refined PGG gives an increase of 22% in core OGCs at a 95% threshold but an increase of 111% in core OGCs at a 100% threshold. When even a single misannotated gene drops an OGC below core at the 100% threshold consistent annotation is crucial.

**Table 2.** Pan-genome graph statistics for *B. subtilis* and *E. coli.*

PGG Statistic	*B. subtilis* original PGG	*B. subtilis* refined PGG	*E. coli* original PGG	*E. coli* refined PGG
Size 1 OGCs	4434	3231	87423	27273
Shared (size>1) OGCs	8174	8204	68970	48129
# of genes in shared OGCs	463311	487562	5039502	5610683
Core OGCs (95%)	3558	3778	2968	3631
Core OGCs (99%)	3356	3604	2168	2992
**Core OGCs (100%)**	**3072**	**3419**	**713**	**1501**
Size 1 edges	7282	5479	153199	67566
Shared edges	9755	9433	99284	67248
Edge instances in shared edges	460452	485177	4970823	5567497
Core edges (95%)	3230	3520	2218	3124

**Figure 2.**
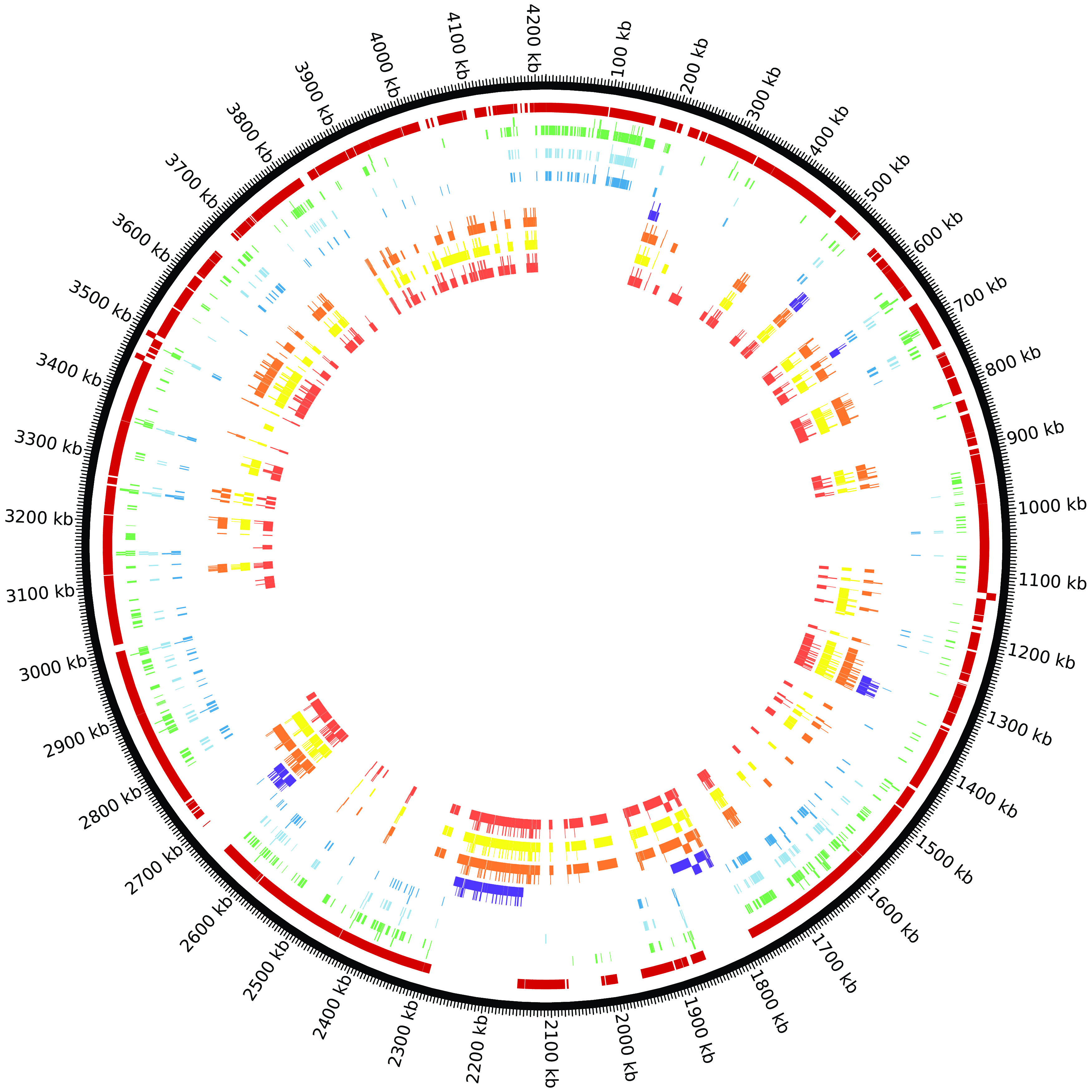
There are eight tracks mapped to the *B. subtilis* reference genome in this Circos figure. Going from the outside to the inside: track 1) core regions (dark red), 2) Minibacillus genes (green), 3) Koo *et al*. ^8^ essential genes (light blue), 4) Kobayashi *et al*. ^7^ essential genes (medium blue), 5) deleted genes in strain delta 6 (dark blue), 6) deleted genes in strain IIG-Bs27-47-24 (orange), 7) deleted genes in strain PG10 (yellow), and 8) deleted genes in strain PS38 (red).

The *B. subtilis* ssp. *subtilis* refined PGG annotates 4654 OGCs for the reference genome (GenBank sequence AL009126.3, BioSample SAMEA3138188, Assembly ASM904v1 **/**GCA_000009045.1) (Supplementary Table 1): 876 (18.8% of OGCs) noncore (<95% of genomes 102 or less), 359 (7.71%) core but not present in all genomes (≥95% and <100% of genomes 103–107), and 3419 (73.5%) core and present in all 108 genomes. For the union of the Koo *et al.* ^8^ and Kobayashi *et al.* ^7^ essential gene data sets there are 305 genes (Supplementary Table 2): 16 (5.25%) noncore (≤102 genomes), 2 (0.656%) core but not all (103–107 genomes), and 287 (94.1%) core all (108 genomes). This shows that most essential genes in *B. subtilis* ssp. *subtilis* are encompassed by core OGCs/regions. There are 258 core regions for *B. subtilis* (Supplementary Table 3). The 289 essential genes which are core OGCs are contained in only 63 of these regions. These 289 essential genes are not evenly distributed in these 63 regions ( *e.g.* 46 are in core region 3). Similarly, the 16 essential genes in non-core regions (the regions between core regions) are contained in only seven non-core regions with eight genes in the non-core region between core regions 206 and 207 ( Figure 2). A table of all *B. subtilis* genes mapped to the reference genome is provided in Supplementary Table 1.

The Reuß *et al.* ^3^ data set for *MiniBacillus* has 523 protein coding and 119 RNA genes predicted to be necessary for a minimal *B. subtilis.* For the 523 protein coding genes: 18 are noncore (≤102 genomes), 16 are core but not in all genomes (one in 105, one in 106, 14 in 107 genomes), and 489 are in all 108 genomes (Supplementary Table 1). They include all 30 rRNA and 86 tRNA genes from the reference genome as well as three “misc” RNA genes in *MiniBacillus.* The three misc RNA genes are present in all 108 genomes. In all likelihood, the 10 copies of the 16S-23S-5S RNA operon are not required but it is safer for robust growth not to delete any of them. Likewise, for the tRNA genes where many are redundant. For the 30 rRNA genes: six are noncore (92–102 genomes), 16 are core but not in all genomes (103–107), and eight are in all 108 genomes. It is clearly possible that some of these strains are dispensing with some of the RNA operons but at most this is happening rarely reinforcing the decision not to remove any from *MiniBacillus.* In addition, some of the missing RNA operon genes may be due to incorrect assembly of the two sets of tandem RNA operons (one a two-unit tandem and one a three-unit tandem) as large tandem repeats can be problematic for assemblers. All the rRNA genes in fewer than 106 genomes are in the tandem rRNA operons (Supplementary Table 4). Of course, the tandem rRNA operons are the most likely to be deleted via recombination as well. For the 86 tRNA genes: 13 are noncore (100–102 genomes), 18 are core but not in all genomes (103–107), and 55 are in all 108 genomes. Retaining all the tRNA genes in *MiniBacillus* also seems to be the correct decision as strains rarely dispose of the tRNA genes.

Both the experimentally determined essential genes and the predicted core OGCs/regions are important data for genome engineering. They both indicate regions that should not be deleted without careful consideration. The noncore regions also indicate where the bacterium is more likely to tolerate engineered insertions. As a validation of our method and how to interpret the results our method produces it is important to understand why 16 essential genes are in noncore regions.

For *B. subtilis*, both Kobayashi *et al.* ^7^ and Koo *et al.* ^8^ used similar single knockout methods to determine “essential” protein-coding genes when grown in LB at 37°C. Koo *et al.* ^8^ identified 257 essential genes while Kobayashi *et al.* ^7^ identified 271 essential genes. The union of these two sets results in 305 essential genes (Supplementary Table 2). The Koo *et al.* ^8^ data set has 257 genes. The Kobayashi *et al.* ^7^ data set has 271 genes. There are 223 genes in common between the two data sets. 48 genes are only in the Kobayashi *et al.* ^7^ data set. 34 genes are only in the Koo *et al.* ^8^ data set. The Kobayashi *et al.* ^7^ data set has been refined with time: ^36^ “Of the original 271 genes, 31 were shown to be non-essential in recent studies. Moreover, 21 new genes (19 protein-coding genes and two RNA-coding genes) were added to the list. Thus, 261 genes encoding 259 proteins and two RNAs are regarded as being essential today”. This list of 259 protein-coding genes is more consistent with the more recent Koo *et al.* ^8^ data set. The 305 genes found in either data set were mapped to the PGG OGCs using the RefSeq genome NC_000964.3 (BioSample SAMEA3138188). Interestingly through this mapping, 16 of the essential genes were not identified as core OGCs (two more essential genes were core OGCs but not present in all 108 genomes). For the 18 essential genes not present in all 108 genomes (Supplementary Table 5), 12 are in both data sets and six are only in the Koo *et al.* ^8^ data set. We believe only 11 of the 18 genes are truly essential. Gene *wapI/yxxG* (OGC 4769 present in 39 of 108 genomes) is an antitoxin for the *wapA* toxin gene which is adjacent to it (present in 85 of 108 genomes). ^37^ Gene *rttF/yqcF* (OGC 4590 present in 46 of 108 genomes) and gene *rtbE/yxxD* (OGC 4772 present in 53 of 108 genomes) are also the antitoxin of a cognate toxin-antitoxin pair. ^38^ Gene *yezG* (OGC 4411 present in 43 of 108 genomes) is also the toxin for a cognate toxin–antitoxin pair. ^39^ Gene *sknR/yqaE* (OGC 4643 present in 34 of 108 genomes) is part of a phage-like region which, if removed would still allow *B. subtilis* to remain viable ^12^ possibly because it is another antitoxin or similar mechanism. Genes *bsuMA/ydiO* (OGC 4838 present in 24 of 108 genomes) and *bsuMB/ydiP* (OGC 4839 present in 24 of 108 genomes) are part of a prophage region of about 15 genes in 48 genomes which includes *ydiR* and *ydiS* which are type-2 restriction enzymes. These are not essential genes, but they are essential if the restriction enzymes are present. ^40^ We are not the first to notice these issues with experimentally determined essential genes indicated by our references above. In their review, Commichau *et al.* ^36^ referred to these as "protective essential genes." In fact, Koo *et al.* ^8^ also addressed this in their paper: “Of the 257 genes essential in LB medium, 30 are not essential in some other growth condition or genomic context...LB may have an insufficient amount of particular compounds; *e.g.*, the *ylaN* mutant requires a higher amount of iron than that present in LB … or may lack a compound that could bypass the need for that gene product; *e.g.*, *eno, pgm, gapA*, and *alrA* … Some gene products are essential only at high growth rates typical of LB at 37°C ( *smc* and *scpA* …), and these may not be essential in the natural soil environment where *B. subtilis* grows slower. Finally, some genes are non-essential in specific genetic backgrounds, *e.g.*, antitoxins can be deleted in strains lacking their cognate toxin gene”.

**Figure 3.**
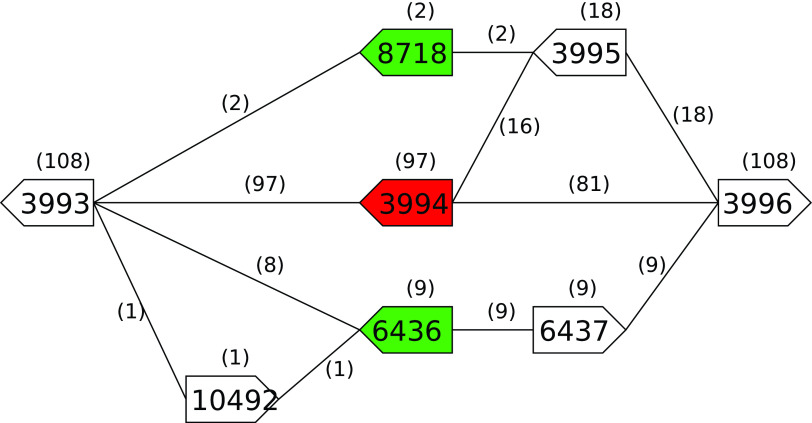
Region of the *B. subtillis* refined PGG encompassing the variation in the *rodA* gene across the pan-genome. OGC 3994 (red) contains the rodA gene from the reference strain. The medoid sequences of OGCs 6436 and 8718 (green) have RefSeq annotations of *rodA* and full-length homology below our 90% threshold to the medoid sequence for OGC 3994. The arrow boxes represent OGCs with gene directionality indicated by the 5′ end being flat and the 3′ end being pointed. Numbers above boxes and edges are the number of genomes the OGC or edge are in.

Another eight essential non-core genes are involved in wall teichoic acid (WTA) biosynthesis: Genes *tuaB* (OGC 4729 present in 85 of 108 genomes), *mnaA/yvyH* (OGC 4735 present in 84 of 108 genomes), *tagH* (OGC 4744 present in 84 of 108 genomes), *tagG* (OGC 4745 present in 35 of 108 genomes), *tagF* (OGC 4746 present in 35 of 108 genomes), *tagD* (OGC 4748 present in 35 of 108 genomes), *tagA* (OGC 4749 present in 35 of 108 genomes) and *tagB* (OGC 4750 present in 35 of 108 genomes). The WTA genes are involved in production of anionic glycopolymers required for consistent cell shape and division. ^41^ The WTA genes are part of a 31 gene region which has been shown to be dispensable ^42^ but results in malformed cells with poor growth properties. Gene *rodA* (OGC 3994 present in 97 of 108 genomes) appears to be the exception as it is asserted to be essential for maintaining a rod shape and preventing spherical cells which lyse. ^43^ Kobayashi *et al.* ^7^ stated: “Ten essential genes are involved in cell shape and division. Septum formation requires seven (ftsA, L, W, and Z, divIB and C, and pbpB …), whereas cell shape requires three (rodA, and mreB and C).” Interestingly, genes *ftsZ* (OGC 1675 present in 105 of 108 genomes) and *pbpB* (OGC 1662 present in 107 of 108 genomes) while considered core, using our 95% of genomes definition are the only core OGCs not present in all 108 genomes. We investigated these 11 genes further to understand why essential genes did not appear to be core OGCs. By examining the PGG we discovered that alternate OGCs with homology to the essential genes had replaced the essential genes. Gene *pbpB* (OGC 1662 in 107 genomes) is replaced in the one remaining genome by OGC 7120 which is also annotated as *pbpB.* Gene *ftsZ* (OGC 1675 in 105 genomes) is replaced in three genomes by a four gene insertion of OGCs 8068, 8300, 8069, and 8070 where both 8068 and 8070 are annotated as *ftsZ.* Gene *rodA* (OGC 3994 in 97 genomes) is replaced by either: OGC 8718 (two genomes) or OGCs 10492, 6436, and 6437 (one genome) or OGCs 6436 and 6437 (eight genomes) where 8718 and 6436 are annotated as *rodA.* As an illustrative example for *rodA*, Figure 3 shows how this is represented in the PGG. The medoid sequences for OGCs 6436 (A4A60_RS20560), and 8718 (C7M30_RS12210) have full length homology to the medoid sequence for rodA (OGC 3994, ETA10_RS20040) with 66% nucleotide /65% peptide and 83% nucleotide/85% peptide identity respectively. For *B. subtilis* ssp. *spizizenii* strain W23, poly (ribitol phosphate) is the main teichoic acid ^44^ and this was thought to distinguish ssp. *spizizenii* from ssp. *subtilis* whose type strain 168 has poly (glycerol phosphate) as the main teichoic acid. Further study found that the ribitol/glycerol distinction does not distinguish between *spizizenii* and *subtilis* subspecies ^45^ but rather either subspecies can contain one or the other. Our PGG confirms this and in fact finds six distinct variants of the WTA region. For example the *tagD* gene (OGC 4748 in 35 genomes) has been replaced by multiple orthologs with the same annotation: OGC 3746 (23 genomes), OGC 5431 (43 genomes), OGC 6915 (two genomes), OGC 7624 (three genomes), and OGC 8731 (one genome). The variation of the WTA region in *B. subtilis* will be the focus of a future paper. ^46^

For the 34 protein coding genes from *MiniBacillus* ^3^ which were not in all 108 genomes (Supplementary Table 6), 10 were already discussed above as to why they were essential but not core. The seven essential genes previously shown to be protective essential genes are as expected not in the *MiniBacillus* data set. The noncore *tuaB* gene was essential in both data sets but not included in *MiniBacillus.* This leaves 24 MiniBacillus protein coding genes which are noncore and unexplained. The tagU and gtaB genes are part of the WTA cassette discussed above. The four *fecC-F* (also called *yfmC-F*) genes form a cassette and are in 98 genomes. From Reuß *et al.*: ^3^ “For iron uptake, the minimal cell should possess the EfeUO system for elemental iron uptake and the iron-citrate ABC transporter YhfQ-YfmCDEF (136, 137).” ( *yhfQ* is present in all 108 genomes) but no alternate mechanism is specified. The seven *purEKBCSQL* genes form a cassette and are in 107 genomes. These genes are involved in purine biosynthesis (see Figure 5 in Reuß *et al.* ^3^) and it is not clear what alternative could be used. The *guaA* gene is involved in nucleotide biosynthesis downstream of purine biosynthesis (see Figure 5 in Reub *et al.* ^3^) present in 107 genomes. The *mntH* gene is a manganese transporter (see Figure 2 in Reuß *et al.* ^3^) present in 106 genomes. The *rlmCD* gene is an rRNA methyltransferase present in 107 genomes. The *lytE*, and *ponA* genes are in 107 genomes. The *pbpB* gene was essential as discussed above and in 107 genomes. The *pbpA* gene is in 50 genomes. From Reuß *et al.*: ^3^ “For the minimal cell, we have selected penicillin-binding proteins 1 (PonA), 2B (PbpB), and 2A (PbpA) and the autolysins LytE and LytF. As outlined above, this selection was made according to their expression profiles and the dependence on other proteins. As an example, there is a functional paralog of LytE, CwlO. For the activity of CwlO, *B. subtilis* also needs the ABC transporter FtsEX and the small protein Mbl. Thus, the choice of LytE allowed a smaller number of genes.”. Interestingly, genes *cwlO*, *ftsE*, *ftsX*, and *mbl* are in all 108 genomes. The *yitI* gene is in 107 genomes. From Reuß *et al.*: ^3^ “Moreover, based on our own experimental data and those of colleagues, YitI, YitW, and YqhY are important for viability (P. Dos Santos, personal communication; our unpublished results).”. The *yoaE* gene is a formate dehydrogenase present in 89 genomes. The *thyB* gene is thymidylate synthase B present in 70 genomes. The *rpoE* gene is in 107 genomes. From Reub *et al.*: ^3^ “Moreover, we have included the RNA polymerase-interacting protein HelD and the nonessential delta subunit (RpoE). HelD binding stimulates transcription in an RpoE-dependent manner, suggesting that these two accessory proteins are important to allow rapid growth (59, 60).”. The *hutM* gene is a histidine permease present in 90 genomes. *MiniBacillus* does not include the adjacent *hutPHUIG* genes which are in 88-91 genomes probably indicating a cassette of genes which interact.

We looked at how our OGGs intersected with the gene deletions from *B. subtilis* strains delta 6, IIG-Bs27-47-24, PG10, and PS38 from Reuß *et al.*’s ^3^ Supplemental Table S1. Strains PG10 and PS38 were derived from strain IIG-Bs27-47-24 which in turn was derived from strain delta 6. This means all deletions in delta 6 are present in the other strains, and all deletions in IIG-Bs27-47-24 are present in PG10 and PS38. For delta 6, most of the deleted genes are noncore which would be expected since most of the deleted regions were phage/prophage regions ( Table 3). For additional deletions to IIG-Bs27-47-24, almost a quarter of the deleted genes are noncore which would again be expected as more prophage and horizontally transferred regions were intentionally targeted but now more core genes were deleted based on core functionality deemed not to be essential for laboratory growth such as sporulation ( Table 3). For additional deletions to PG10 and PS38, most deleted genes were core as most of the obviously horizontally transferred regions had already been deleted ( Table 3). While pan-genome analysis was not used to select the deleted regions, we believe it could have provided strong evidence to support the deletion of the noncore genes/regions which were deleted. In addition, it could be used to suggest further deletions. There are nine noncore regions which contain seven or more noncore genes which have not yet been deleted in any of these strains ( Table 4). The largest of these regions contains the WTA genes cassette we discussed above and is not a good candidate for deletion. By examining the refined PGG at these regions it is straightforward to determine if there are alternate OGC choices for the region that in sum designate the region as likely to be core as we showed in Figure 3.

**Table 3.** The number of deleted genes from *B. subtilis* reduced strains which are noncore versus core.

Strains	Number of noncore deleted genes	Number of core deleted genes
delta 6, IIG-Bs27-47-24, PG10, PS38	340	46
IIG-Bs27-47-24, PG10, PS38	232	792
PG10, PS38	7	57
PG10	14	78
PS38	15	129

**Table 4.** Large noncore regions which have not been deleted from any of the strains delta 6, IIG-Bs27-47-24, or PG10, PS38.

First gene	Last gene	Number of noncore genes in region	Number of core genes in region	Alternate genes in refined PGG
BSU04270, *epsJ*,OGC4339	BSU04320, *kimA*,OGC4344	7	0	no
BSU05040, *yddN*,OGC4348	BSU05110, *sufLC*,OGC579	10	0	yes
BSU07440, *yfmK*,OGC4418	BSU07550, *yflT*,OGC4427	10	0	no
BSU11910, *yjcM*,OGC4457	BSU11990, *yjdB*,OGC4465	10	1	yes
BSU18940, *yobHm*,OGC4568	BSU19000, *rttL*,OGC2078	10	0	yes
BSU29280, *ytnM*,OGC4678	BSU29400, *ascR*,OGC4689	13	0	no
BSU35550, *tuaG*,OGC4724	BSU35770, *tagC*,OGC4751	28	0	yes
BSU37220, *ywjB*,OGC3904	BSU37320, *narK*,OGC3915	11	0	yes
BSU39850, *yxbF*,OGC4783	BSU39920, *asnH*,OGC4790	9	0	yes

To show that our method produces significantly different results than previous methods we compared our pan-genome analysis to the very recent work on a *B. subtilis* pan-genome by Wu *et al.* ^47^ While the focus of Wu *et al.* ^47^ was on determining which genomes should be excluded from a species/subspecies pan-genome based on “incorrectly classified *Bacillus* subspecies strains, phylogenetically distinct strains, engineered genome-reduced strains, chimeric strains, strains with a large number of unique genes or a large proportion of pseudogenes, and multiple clonal strains”, their analysis focused on how this affected the determination of core OGCs. We compared our core OGC set to theirs for the reference genome. Wu *et al.* ^47^ discussed two pan-genome data sets: “old (89 strains) and new (153 strains)”. We compared to the new data set which is more recent and more comparable to our pan-genome of 108 strains (Supplementary Table 5). After removing “confounding” strains the new data set had 128 strains. From their Table 1 compared to our Table 2, Wu *et al.* ^47^ have many fewer core OGCs whether defined at 95%, 99%, or 100% both for our original and refined PGGs. We compared their methods to ours to attempt to account for the difference. They also apparently restricted genomes to those available from RefSeq since they mention a RefSeq ID. They did not require the genomes to be considered complete by RefSeq as we did but instead used these criteria: “Among these *B. subtilis* strains, we removed strains whose N base content was greater than 1% of the genomic size (FB6-3,GS 188, SR1), and we removed the chimeric genome BEST7613 with a genome size of 7.6 Mb.”. We used the RefSeq annotation which is generated by a consistent NCBI annotation pipeline. They also tried to ensure consistent annotation: to “ensure the consistency and reliability of the annotation and gene prediction of the genome, we used the program Prokaryotic Genome Annotation System (Prokka)”. We doubt the different annotations from these two established pipelines accounts for many differences in core OGCs. Both methods used a whole genome ANI method to discard outlier genomes. There are multiple differences in our pan-genome approach. First, we used PanOCT and they used Roary. Second, we used all annotated gene features: gene (protein coding), pseudogene, miscRNA, rRNA, and tRNA, whereas they used only protein-coding genes. Finally, and we think most importantly, we iterated over annotating the genomes and PGG refinement to ensure consistent annotation and they did not. To see what impact our choice of all gene features versus just protein-coding genes had we looked at the annotation of core OGCs on the reference genome (Supplementary Table 1). Luckily all 3778 core (95% threshold) OGCs are present in the reference genome. Of these, 3473, 3334, and 3189 are protein coding OGCs at thresholds 95%, 99%, and 100% respectively. All these numbers are still much higher than those reported by Wu *et al.* ^47^ We should note that even though we did not count the 25 core OGCs annotated as pseudogenes in the reference genome, some of the core protein-coding OGCs in the reference genome might be annotated as pseudogenes in other genomes which could impact the Wu *et al.* ^47^ numbers. Roary tends to require near full length gene matches which is why we required PanOCT to only use 90% or longer length matches. The authors chose to limit Roary to 95% identity or higher matches which we think is much too high since the species ANI threshold is 95% and even subspecies ANI threshold of 98% is too close to this threshold given that some genes are more rapidly evolving than others so we used a threshold of 90% or higher identity for matches. Even with our 90% identity threshold some genes such as *rodA*, discussed above, drop below this threshold generating possibly unnecessary branching in the PGG. Of the 128 strain pan-genome from Wu *et al.* ^47^ that we compared to our 108 strain pan-genome, 92 strains were in common with 16 being exclusive to our pan-genome and 36 being exclusive to theirs. Of the 36 strains exclusive to theirs 23 were removed as being redundant at the ANI level by us, 9 were in RefSeq but not complete genomes, and 4 either were never in RefSeq (they do not have RefSeq IDs in their Supplementary Table 3) or no longer are. Interestingly, while 15 of the 16 genomes exclusive to ours are just more recent strains to RefSeq, one strain, D12-5, was used by us but discarded by them. They discarded D12-5 because “BS155 and D12-5 possess the largest proportion of pseudogenes (37.96% and 11.32%) among the *B. subtilis* strains” and for D12-5 they indicated this was due to a large number of frameshifts. Pseudogenes due to frameshifts are often an indication of lower quality assembly consensus sequence from using only long reads at lower coverage. Our pan-genome method is resilient to this kind of error profile in the genome due to reannotation of the genomes and PGG refinement whereas other pan-genome methods are not. We believe our higher counts for core protein coding OGCs is correct. To validate this, we compared how many of the 305 essential *B. subtilis* genes are core for both methods. For the 18 genes we discussed above that are essential but not in all 108 genomes of our PGG, 2 are core at 95% and 1 is core at 99%; whereas, for Wu *et al.* ^47^ 2 are core at 95%, 2 are core at 99%, and 1 is core at 100%. The only significant difference for these 18 genes is that *ftsZ* is in 95% (105) of our pan-genome and 100% of theirs. The Wu *et al.* ^47^ pan-genome misses many additional essential genes which ours does not: 28, 39, and 47 for 95%, 99%, and 100% thresholds respectively.

**Figure 4.**
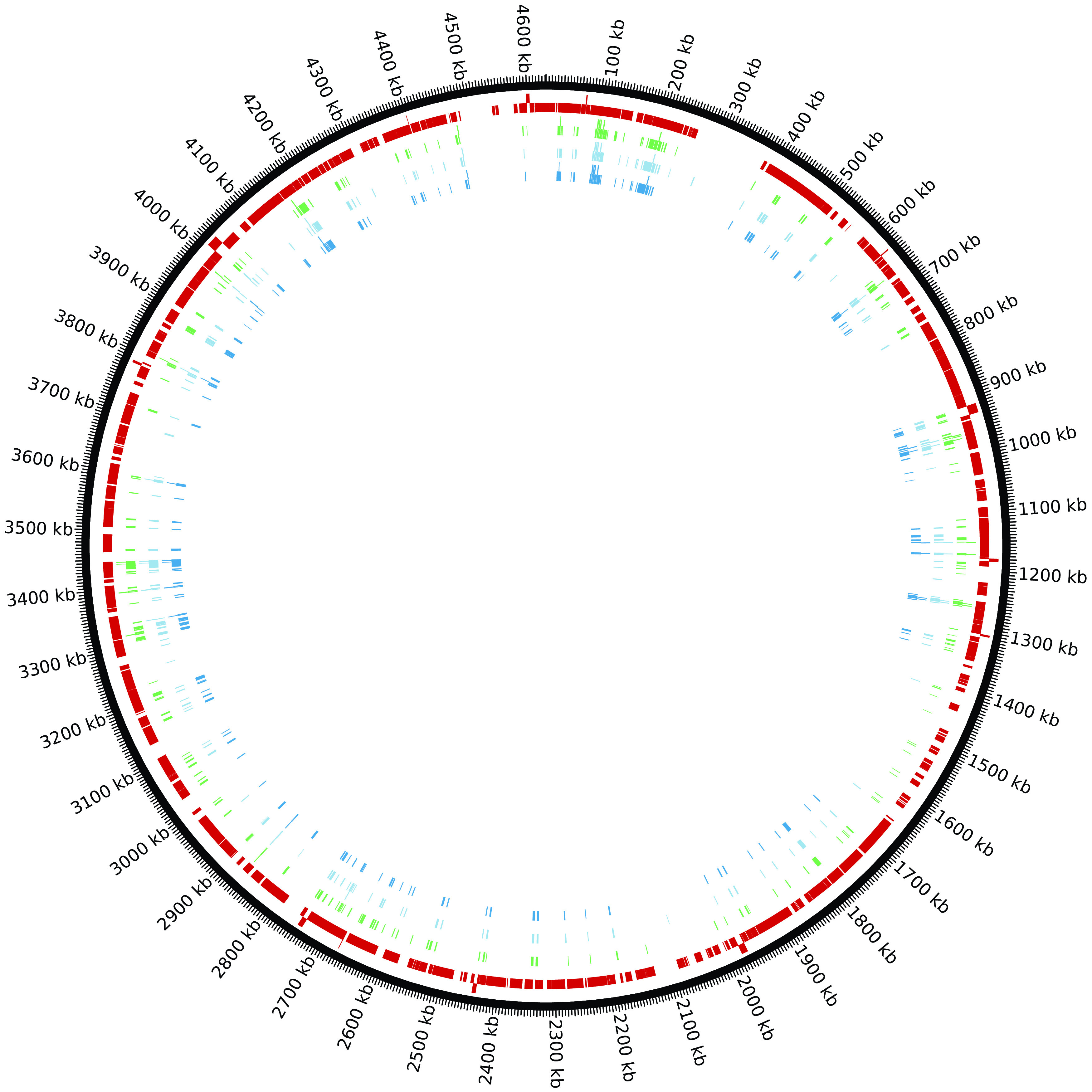
There are four tracks mapped to the *E. coli* reference genome in this Circos figure. Going from the outside to the inside: track 1) core regions (dark red), 2) TraDis essential genes (green), 3) Keio essential genes (light blue), and 4) PEC essential genes (medium blue).

For *E. coli*, Goodall *et al.* ^9^ determined *E. coli* essential genes using an analysis of transposon insertion events (TraDIS). The results of their study and two other studies, the Keio collection ^10^ and the Profiling of the *E. coli* Chromosome (PEC) ^11^ were captured in Table S2 of Goodall *et al.* ^9^ Of the 414 genes with overlap between these studies, the 248 essential genes in common for all three studies are all core OGCs ( Figure 4 and Supplementary Table 7). This set of 248 essential genes should be the highest quality predictions as determined by all three studies and confirms our assertion that essential genes should almost always be core OGCs. The next highest quality set of essential gene predictions is the 45 essential genes where two of the three studies agree which 41 are core OGCs: for Keio–PEC, 15 of 16 are core OGCs; for TraDIS–Keio, eight of 11 are core OGCs; and for TraDIS–PEC, 18 of 18 are core OGCs (Supplementary Table 7). The lowest quality set of essential gene predictions is the 121 essential genes where only one study agrees which 89 are core OGCs: for Keio only, 12 of 22 are core OGCs; for PEC only, 18 of 18 are core OGCs; and for TraDIS only, 59 of 81 are core OGCs (Supplementary Table 7). One of the noncore essential genes present in two studies (TraDIS–Keio), *racR,* is probably a toxin suppressor which is not essential in the absence of the toxins. Bindal *et al.* ^48^ noted, “We further show that both YdaS and YdaT can act independently as toxins and that RacR serves to counteract the toxicity by tightly downregulating the expression of these toxins”. The *racR* gene is found in only 106 of the 971 genomes in the *E. coli* PGG, whereas *ydaS* and *ydaT* are found in 106 and 150 genomes respectively, perhaps arguing that *ydaS* is the key toxin gene. This recapitulates the pattern we observed in *B. subtilis* where toxin suppressor genes are only essential in the presence of toxin genes. Similarly, the *dicA* gene (TraDIS–Keio) can be deleted if the *dicB* gene is also deleted. Kato *et al.* ^49^ noted: “The *dicA* gene encoding a repressor of a cell division inhibitor was deleted in our study with the *dicB*, the inhibitor gene”. There are 521 core regions for *E. coli* (Supplementary Table 8). The 378 essential genes which are core OGCs are contained in only 133 of these regions. These 378 essential genes are not evenly distributed in these 133 regions ( *e.g.*, 27 are in core region 362). Similarly, the 36 essential genes in non-core regions (the regions between core regions) are contained in only 23 non-core regions with four in the non-core region between core regions 152 and 153. A table of all *E. coli* genes mapped to the reference is provided in Supplementary Table 9.

Yang *et al.* ^50^ presented a similar pan-genome analysis for 491 *E. coli* strains. There were 420 strains in common between the Yang *et al.* ^50^ 491 strain pan-genome and our 971 strain pan-genome (Supplementary Table 10). Our pan-genome included *Shigella* species (see Methods) which Yang *et al.* ^50^ did not. This added diversity of our pan-genome should reduce the number of core OGCs. Likewise, the much larger number of strains in our pan-genome should reduce the number of core OGCs. Yang *et al.* ^50^ report 867 core protein-coding genes presumably at a 100% threshold although this is not explicitly stated. For our refined PGG, we had 1501 core OGCs at the 100% threshold. We include all genes in our OGCs but 1234 of the 1501 core OGCs are protein coding at the 100% threshold. Yang *et al.* ^50^ did not provide a table of their core genes for sake of comparison, however we expect for the same reasons as for our more detailed analysis of the *B. subtilis* pan-genome that our set of core OGCs is more complete. Yang *et al.* ^50^ reported that their core genes included 243 essential genes from the DEG database ^51^ which contains essential genes from many studies but did not provide a table of these genes. Yang *et al.* ^50^ also reference two essential gene studies one by Gerdes *et al.* ^52^ and one by Baba *et al.* ^10^ which was one of the three studies we used (Keio). In the DEG database the Gerdes *et al.* ^52^ study has 609 essential genes, and the Baba *et al.* ^10^ study has 296 essential genes. Our version of the Baba *et al.* ^10^ study we called Keio had 297 essential genes of which 218 were core OGCs at the 100% threshold. For the union of the three studies we compared against, we had 289 essential genes out of 414 which were core OGCs at the 100% threshold. It is unclear whether the 243 core essential genes Yang *et al.* ^50^ reported were from the Baba *et al.* ^10^ study, the Gerdes *et al.* ^52^ study, or the union of the two studies. Given the much lower number of core genes for the Yang *et al.* ^50^ core genes compared with our core OGCs, we believe that Yang *et al.* ^50^ used the union of essential genes from the Baba *et al.* ^10^ and Gerdes *et al.* ^52^ studies.

There is of course no “gold standard” that provides a 100% correct set of core regions/genes for a pan-genome/species. When comparing our method to others, this leaves only indirect measures of accuracy. We compared our method versus two other recent core gene determinations for *Bacillus subtilis *and *Escherichia coli *and showed that our method was superior using coverage of essential genes by core genes as an indirect measure. We also showed that the PGG allowed for a detailed analysis of exceptions such as when an OGC is replaced by a more distant ortholog.

## Discussion

For the purpose of biological engineering, determining the set of core regions for a given species is critical as changes to these regions should be expected to reduce fitness or be lethal. Core regions indicate parts of the genome that are conserved across evolution within a species. These regions are not necessarily required for survival but presumably confer a fitness advantage and define the characteristic core genotype which produces the core phenotype (lifestyle). Since most essential gene studies are carried out under specific static laboratory growth conditions, genes which would normally be essential for a species across a diverse set of dynamic environmental conditions might not be discovered ( *e.g.*, necessary for fluctuating temperatures). Correspondingly, genes required to out compete rival organisms through increased fitness or to evade immune responses might not be found under laboratory conditions are considered facultative essential. ^31^ Core regions, therefore, should be a superset of essential genes in most cases but exceptions might occur for genes which are not needed in a species’ natural niche but are required in a laboratory setting. Another exception would be for genes which are essential for a particular strain but not for other strains due to the presence of compensating non-core genes.

Noncore OGCs/regions which are determined by pan-genome analysis are often horizontally transferred elements, such as phage, prophage, or mobile elements. For industrial applications these regions are dispensable and can even be sources of genome instability. ^3, 12– 14, 53^ While there are other methods for identifying these regions, pan-genome analysis is a reliable complimentary tool. Pan-genome analysis can also reveal enzymatic and other systems/pathways that are present in some strains but not others ^53^ which indicates they can likely be removed. When choosing between retaining alternate systems for essential functions, biological engineers have looked at conservation of those systems across broad taxonomic levels ^3^ as an indication of utility and we believe conservation across the pan-genome should also be considered. When specific genes/systems of known function are being targeted for removal pan-genome analysis is less useful but still good information to have. For instance, Reuß *et al.* ^2^ tried to delete region BSU07710-07820 from *B. subtilis* which was lethal. In this region, six of the 11 OGCs are core but the five noncore genes are adjacent so perhaps region BSU07750-07790 could have been successfully deleted.

Given that we believe pan-genome analysis is a useful complimentary tool for biological engineers, it is important that the pan-genome analysis used be as accurate and helpful as possible. We showed by comparing with other recent pan-genome studies for *B. subtilis* and *E. coli* that our method is more accurate for determining core OGCs/regions as validated by coverage of essential genes. Further, we believe that the PGG is valuable for confirming when noncore OGCs may be compensated for with alternate homologous OGCs at the same relative genomic location performing the same function as we showed in Figure 3. The function of these noncore OGCs may be essential and should be considered appropriately.

Pan-genome studies often capture the diversity of sequenced species but fail to compare gene lists to experimentally validated essential genes lists or the results are confusing. Interestingly in *Mycoplasma*, fewer essential genes were determined with the pan-genome method compared with the laboratory experimental approach. ^54^ In *Pseudomonas*, only one-third of the pan-genome single copy genes had overlap with the essential genes from experimentally reduced genomic studies. ^55^ We showed that the core OGCs/regions from our refined PGG encompass 91% and 95% of the *E. coli* and *B. subtilis* experimentally determined essential gene lists *,* respectively. Both model bacterial species *E. coli* and *B. subtilis* have had many genome reduction studies performed and reviewed elsewhere. ^56^

Experimental verification of the essentiality of computationally predicted core OGCs or regions requires that each strain of the pan-genome study be minimized. However, it is cost prohibitive to do knockout studies on all strains of a pan-genome. One must carefully choose a single genome as a representative of the entire pan-genome for the purpose of verifying the essentiality of core regions and/or the non-essentiality of noncore regions by experimental validation. However, given the diversity of most bacterial species it is unlikely that any one strain completely captures the capabilities of the species in all environmental conditions. Further, while there are clearly core OGCs/regions associated with viability for a species, other core regions probably contribute to a lesser degree to cell viability. For example, for the purpose of biological engineering, changes in these locations may reduce fitness by slowing cell growth.

The use of a PGG for identifying core regions of a bacterium is an automatable, low-cost, rapid, and effective way to evaluate both Gram-negative and Gram-positive bacteria. This method compliments and expands upon the experimental knockout approach by including environmental diversity as a measure of what regions and OGCs are conserved across the species. The approach also overcomes the limitations of knockout studies that are specific to the strains and growth conditions used.

The *B. subtilis* WTA region provides a cautionary note for relying entirely upon core regions to determine what is safe to remove. While most non-core regions involve cassettes of genes which are entirely absent from some strains such as phage regions, sometimes orthologous replacement possibly due to homologous recombination can have functionally equivalent genes appearing to be non-core. A closer examination of the PGG can determine if a region is simply missing from some strains versus being replaced in which case further study may be needed before removal of the region. Of course, in some cases the orthologous replacement does not need to occur at the same location in the genome but that was the case for all instances we examined in *B. subtilis.*

While we showed that almost all essential genes are core OGCs and most are OGCs at the 100% threshold, the exceptions are interesting. We discussed issues such as “protective essential genes” ^36^ (such as toxin/anti-toxin gene pairs) and more distant orthologs not captured in OGCs. We did not discuss genes which might be undergoing gene loss. ^57^ The PGG is well suited to looking at which subset of genomes have suffered a gene loss and possible mechanisms such as gene replacement. The PGG has been used to show which genomic regions tend not to allow insertions of horizontally transferred genes ^19^ and where metabolic cassettes can be swapped. ^53^

## Data Availability

Figshare: Underlying data for ‘A pan-genome method to determine core regions of the *Bacillus subtilis* and *Escherichia coli* genomes’, https://doi.org/10.6084/m9.figshare.15129636.v1. ^58^ This project contains the following underlying data:
• Table 1. Selection of complete genomes for *B. subtilis* and *E. coli* PGGs.
• Table 2. Pan-genome graph statistics for *B. subtilis* and *E. coli.*
• Table 3. The number of deleted genes from *B. subtilis* reduced strains which are noncore versus core. • Table 4. Large noncore regions which have not been deleted from any of the strains delta 6, IIG-Bs27-47-24, or PG10, PS38. Data are available under the terms of the Creative Commons Attribution 4.0 International license (CC BY 4.0). Figshare: Extended data for ‘A pan-genome method to determine core regions of the *Bacillus subtilis* and *Escherichia coli* genomes’, https://doi.org/10.6084/m9.figshare.15129636.v1. ^58^ This project contains the following extended data:
• Supplementary Table 1 • Supplementary Table 2 • Supplementary Table 3 • Supplementary Table 4 • Supplementary Table 5 • Supplementary Table 6 • Supplementary Table 7 • Supplementary Table 8 • Supplementary Table 9 • Supplementary Table 10 Data are available under the terms of the Creative Commons Attribution 4.0 International license (CC BY 4.0).
