# Supplementary material for: A pan-genome method to determine core regions of the Bacillus subtilis and Escherichia coli genomes
Source: F1000Res. 2021 Sep 2;10:286. Originally published 2021 Apr 13. [Version 2] doi: 10.12688/f1000research.51873.2 (PMC8156514; doi:10.12688/f1000research.51873.2)
Supplement: Peer review history

# Peer review: Reviewer response for version 1

**DOI:** 10.5256/f1000research.55083.r84280

**Published:** 2021-05-26

**Authors:** Christos Ouzounis

**Affiliations:** 1 Department of Computer Science, Aristotle University of Thessalonica, Thessalonica, Greece; 2 Centre for Research & Technology Hellas, Thessalonica, Greece

This extensive, complex report provides details about a new methodological approach for the detection of ‘core’ regions of *Bacillus subtilis* and *Escherichia coli*. Core regions are defined within the pan-genome context of conserved genomic loci for the two bacterial species, as a case study. An underlying assumption and implicit goal of the study is that the detected core regions largely correspond to ‘essential’ genes, as those have been determined by independent experimental methodology, with implications for synthetic engineering of bacteria. For both purposes, namely the detection of core regions and the correspondence of those to essential genes, this report is an important contribution, especially as it resolves the connection of core to essential genes. It brings to the forefront the use of pangenome analysis for synthetic biology – a factor that so far has been, to our amazement (!), ignored by biotechnologists. Solid work and a significant contribution to the field.

**Major comments:**

1. A general stylistic observation is that the manuscript is dense, in particular the Introduction and Methods are quite extensive and discursive, the Introduction containing multiple quotes from previous works. While this is not necessarily a bad thing, some details (“in their table 4, etc.” and other quoted phrases from cited papers) could be avoided or better summarized. This level of detail is welcome for experts, but non-experts are at risk to miss the main point and the motivations for this study. A more standard style, perhaps for the first paragraph might be useful, in order to address a wider audience.
2. “While it is possible to define “essential” genes”: the definition of ‘essential’ genes is problematic as it refers to the growth medium and general environmental conditions, as the authors correctly point out. Therefore, ‘essentiality’ is a functional definition. Core (conserved, species-defining) genes, on the other hand, do not rely on environmental factors but evolutionary history, therefore ‘conservation’ is a structural definition. Coupling those is always tricky, however as the authors state early on in their paper, the equivalence between core and essential genes is indeed their primary hypothesis (“We expect that all truly essential genes for the species/subspecies would be a subset of the core OGCs/regions”). This should be more explicitly stated, perhaps in the first paragraph of the Introduction.
3. What advantage is provided by keeping the directionality of OGCs in the PGG? Is this purely a methodological checkpoint, i.e. improve the detection capability by reducing the number of false positive or negative hits, or is it further used in the analysis and interpretation of the results? Needs to be clarified, as it increases the complexity of the pan-genome turning a set into a graph. There is a passage “PGG refinement to ensure consistent annotation”, which alludes to the actual role of PGG.
4. Another general comment connected to the above, esp. major comment 2: the report serves a dual role as a software announcement (update) of JCVI’s pan-genome pipeline software suite, with additional elements and certain conceptual advances, as well as the comparison of the core-vs-essential sets for two of the best studied/sampled species pangenomes. This should be a bit more clearly explained perhaps. The correspondence of core to essential genes is a welcome contribution but may not be the main topic of the manuscript, just a conclusion drawn from the analysis.
5. Following major comment 4: the method does well in identifying core regions and indeed makes a convincing case for an improvement over other methods. Yet, the comparison with essential genes is an addition, but not a comparison against other methods that define core regions. As the authors decided to take this direction, as they improve over their own previous methodology, this point should be qualified appropriately. In other words, the ‘improvement’ can be shown as an incremental step over a previous protocol and explicitly shown that it is validated against ‘essential’ gene sets. If this point is not emphasized, the analysis will be seen as lacking a comparison to another ‘gold-standard’ method (experts know that there is no such thing, yet). Pages 11-12 have some elements of a comparison to another approach, this could be extended by a couple of concluding sentences. A good spot where some concluding remarks can be made might be a short paragraph before the Discussion.

**Minor comments:**

1. In Introduction: “We further define a pan-genome graph (PGG) to be a graph”, this should probably follow the paragraph starting “Here we present a pan-genome based calculation...” ?
2. “For *E. coli* (and *Shigella*) we downloaded 1097 complete genomes”, start a new paragraph? Using subtitles for Methods might also be a good idea, to break down the dense text into digestible sections.
3. Following minor comment 2: a mini table with three columns (filtering step, *B. subtilis*, *E. coli*) and as many rows as the filtering steps used with the number of genomes at each step might be helpful.
4. “used by Goodall” (reference 9? missing).
5. “This is done by blasting” - executing BLAST etc. / “conflicting blast” -> conflicting BLAST...
6. “to not under call core OGCs/edges”, i.e. to reduce the number of potentialy false negatives. Or, increase coverage.
7. for *B. subtilis*: “3419 (73.5%) core and present in all 108 genomes”: this row in Table 1 should be somehow highlighted, perhaps by color or other means -- it is an important part of the study and a key result.
8. a word for missing genes in the context of potential gene loss and the possibility of including them in future steps (see PMID: 12840037 ^1^); this is something we (and possibly others) have been trying to implement for pangenome data, without much success. Something to discuss as a partial explanation for ‘key’ (essential?) missing genes in certain lineages within the species pedigree, perhaps?
9. “For the 34 protein coding genes”... good yet incredibly dense paragraph, a (supplementary) table might help here.
10. Would the PGG implementation also help future studies in synteny analysis/conservation? Maybe a minor point that can be included in the discussion, with appropriate (1-2) references. A concluding short paragraph following the current one with the WTA region might be a good way to wrap up.

Is the work clearly and accurately presented and does it cite the current literature?

Yes

If applicable, is the statistical analysis and its interpretation appropriate?

Yes

Are all the source data underlying the results available to ensure full reproducibility?

Yes

Is the study design appropriate and is the work technically sound?

Yes

Are the conclusions drawn adequately supported by the results?

Yes

Are sufficient details of methods and analysis provided to allow replication by others?

Yes

Reviewer Expertise:

Computational Biology, Biological Computation, Systems Biomedicine, Bioinformatics, Protein Structure

I confirm that I have read this submission and believe that I have an appropriate level of expertise to confirm that it is of an acceptable scientific standard.

## References

[1] : The balance of driving forces during genome evolution in prokaryotes.*Genome Res*.2003;13(7) : 10.1101/gr.10926031589-9410.1101/gr.109260312840037PMC403731

# Response

**Published:** 2021-08-09

**Authors:** Granger Sutton

**Affiliations:** J. Craig Venter Institute, USA

We thank the reviewer for the thoughtful comments and have tried to respond to all of the suggestions including the new table and supplementary table in version two of our manuscript.

# Peer review: Reviewer response for version 1

**DOI:** 10.5256/f1000research.55083.r83244

**Published:** 2021-04-28

**Authors:** David Ussery, Kaleb Abram

**Affiliations:** 1 Programming Associate, DBMI, University of Arkansas for Medical Sciences, Little Rock, Arkansas, USA; 2 Department of Biomedical Informatics, University of Arkansas for Medical Sciences, Little Rock, AR, USA

**Introduction:**

 The authors provide an adequate background literature detailing attempts by others in the field to produce minimal genomes. A variety of approaches are covered and drawbacks to these attempts are mentioned. The authors also provide sufficient explanation of the need for their approach in addition to experimental approaches. A good overview of their pan-genome graph approach is presented, along with the reasoning for their design choices for the graph.

 Towards end of first paragraph in Introduction - should be _G_ram-negative (name for the Danish microbiologist, Hans Christian Gram)

 Last sentence in last paragraph in Introduction - "Our method builds directly upon our previous pan-genome work and includes several improvements: 1) being able to _automatically_ use only complete high-quality genomes..."  Surely the previous methods could also have used only complete high-quality genomes as input?  My understanding is that the advantage of this new method is that it's now taking steps to ensure that 'bad genomes' are filtered out, and only the 'high-quality' ones are left....

**Methods:**

 Figure 1, 2nd line: "Compute genome ANI using Mash". This doesn't make sense, as ANI and Mash are different approaches. Mash does not estimate ANI (as it is a distance). Unless the authors took the distance and subtracted it from 1 before multiplying by 100, they do not have an approximate ANI (see fastANI paper [PMID: 30504855] ^1^ or the Mash paper [their ref. 34] where Mash and ANI methods are compared). Further, the authors state they use type strains and ANI (presumably using the Mash derived approximation which is not ANI) to remove very closely related strains but do not specify what criteria/value was used to determine very closely related strains. Since the authors chose to use a program (GGRaSP) that uses ANI matrixes as the input, it can be assumed that either ANI values were calculated by an unspecified method, or they used transformed Mash values to approximate ANI and need to specify this transformation earlier in the methods. Either way, this should be clearly stated in the methods section, and not leave the reviewer to guess how this might have been done.

 “The 132 genomes were reduced to 109 after removing…” – it is unclear what the condition for removal was.  It would be helpful if this was explicitly stated (presumably an approximate ANI value between 95.73% and 97.28%). Also the authors state the minimum ANI between B. subtilis was 97.28% and the maximum ANI of any of the 11 other genomes to the 132 was 95.73%. The 11 genomes referenced here are unclear and the maximum ANI for the 132 is not provided. It is important to clearly bound their values, in order to enable comparison to other studies. For E. coli the parameters used to remove redundancy need to be explicitly stated and how the groups are collapsed (i.e. genomes A to genome B has 99% ANI value, which genomes is removed and which genome is retained?). The authors should explicitly state why they added 2 redundant genomes to the E. coli dataset but did not do similar additions for B. subtilis. While the PGG approach seems fairly good, the heavy reliance on RefSeq annotations could be problematic for other species.

**Results:**

 The results shown in Table 1, and the bottom line is that for both B. subtilis and E. coli, the refined cores are a bit larger (and contain a larger fraction of 'essential genes' for the species).  The E. coli core is about a third larger, going from 2200 to 3100.  The latter number (3100) seems to be more consistent with what's expected for E. coli, based on many different experiments - historically, there has always been roughly 3000 E. coli genes.  So from this perspective, 2218 genes seems a bit too small (and also some of the 'essential genes' were missing from the core.)

 I'm curious as to whether a non-RefSeq gene annotation tool (for example, Prokka) be utilized to improve the consistency of gene calls? The specific results with number breakdowns are very confusing to read on a first pass and require very careful reading to understand the somewhat odd notation being used. This should be cleaned up to enhance readability. 

 Figure 2 should have a color key containing color to corresponding track to increase readability of this figure. (The same thing for Figure 4 for consistency.) 

**Discussion:**

 The discussion surrounding the issue of lab conditions and core regions is a good. In addition, the discussion around noncore OGCs/regions also shows how the proposed pan-genome analysis could be used to identify noncore regions that could be removed that experimental results have been unable to identify. It might have been good to have a brief discussion of the phylogroup-specific cores in E. coli [see PMID: 33500552 ^2^ - disclaimer - this is a recent publication from our group.]

 The discussion section overall provides a good wrap up to the paper and summarizes how the PGG approach can be leveraged and the benefits from utilizing this approach.

Is the work clearly and accurately presented and does it cite the current literature?

Yes

If applicable, is the statistical analysis and its interpretation appropriate?

Yes

Are all the source data underlying the results available to ensure full reproducibility?

Yes

Is the study design appropriate and is the work technically sound?

Yes

Are the conclusions drawn adequately supported by the results?

Yes

Are sufficient details of methods and analysis provided to allow replication by others?

Yes

Reviewer Expertise:

Comparative genomics

We confirm that we have read this submission and believe that we have an appropriate level of expertise to confirm that it is of an acceptable scientific standard.

## References

[1] : High throughput ANI analysis of 90K prokaryotic genomes reveals clear species boundaries. *Nature Communications*.2018;9(1) : 10.1038/s41467-018-07641-910.1038/s41467-018-07641-9PMC626947830504855

[2] : Mash-based analyses of Escherichia coli genomes reveal 14 distinct phylogroups.*Commun Biol*.2021;4(1) : 10.1038/s42003-020-01626-511710.1038/s42003-020-01626-533500552PMC7838162

# Response

**Published:** 2021-08-09

**Authors:** Granger Sutton

**Affiliations:** J. Craig Venter Institute, USA

We thank the reviewers for their thoughtful comments and have attempted to address all of the suggestions in version 2 of our manuscript.
